# Use of SNP chips to detect rare pathogenic variants: retrospective, population based diagnostic evaluation

**DOI:** 10.1136/bmj.n792

**Published:** 2021-03-22

This paper by Weedon and colleagues (BMJ 2021;372:n214, doi:10.1136/bmj.n214) should have a CC-BY open access licence rather than the CC-BY-NC licence with which it was originally published. The online version has been amended.

